# The effect of deep vein thrombosis on major adverse limb events in diabetic patients: a nationwide retrospective cohort study

**DOI:** 10.1038/s41598-021-87461-y

**Published:** 2021-04-13

**Authors:** Po-Chang Wang, Tien-Hsing Chen, Chang-Min Chung, Mei-Yen Chen, Jung-Jung Chang, Yu-Sheng Lin, Pao-Hsien Chu, Yun-Shing Peng, Ming-Shyan Lin

**Affiliations:** 1 Division of Cardiology, Department of Internal Medicine, Chang Gung Memorial Hospital, No. 6, Sec. West Chai-Pu Road, Pu-TZ City, 61363 Chiayi, Taiwan; 2 Division of Cardiology, Department of Internal Medicine, Chang Gung Memorial Hospital, Keelung, Taiwan; 3 Department of Nursing, Chang Gung University of Science and Technology, Chiayi, Taiwan; 4 Department of Nursing, Chang Gung University, Taoyuan, Taiwan; 5 Division of Cardiology, Department of Internal Medicine, Linkou Medical Center, Chang Gung Memorial Hospital, Taoyuan, Taiwan; 6 School of Medicine, Chang Gung University, Taoyuan, Taiwan; 7 Division of Endocrinology and Metabolism, Department of Internal Medicine, Chang Gung Memorial Hospital, Chiayi, Taiwan; 8 Graduate Institute of Clinical Medical Sciences, College of Medicine, Chang Gung University, Taoyuan, Taiwan

**Keywords:** Cardiology, Diseases, Medical research

## Abstract

Little is known about the association between deep vein thrombosis (DVT) and arterial complications in patients with type 2 diabetes (T2DM). The aim of this retrospective cohort study was to assess the influence of prior DVT on major adverse limb events (MALEs) and major adverse cardiovascular events (MACEs) in T2DM. A total of 1,628,675 patients with T2DM with or without a history of DVT from 2001 to 2013 were identified in the National Health Insurance Research Database of Taiwan. Before matching, the patients in the DVT group (n = 2020) were older than the control group (66.3 vs. 58.3 years). Patients in the DVT group were more likely to be female than the control group (54.3% vs. 47.5%). Before matching, the DVT group had higher prevalence of most comorbidities, more prescription of antiplatelet, antihypertensive agents and insulins, but less prescription of metformin and sulfonylurea. During a mean follow-up of 5.2 years (standard deviation: 3.9 years), the matched DVT group (n = 2017) have a significantly increased risk of MALE (8.4% vs. 5.2%; subdistribution hazard ratio [SHR] 1.60, 95% CI 1.34–1.90), foot ulcer (5.2% vs. 2.6%, SHR 1.96, 95% CI 1.57–2.45), gangrene (3.4% vs. 2.3%, SHR 1.44, 95% CI 1.10–1.90) and amputation (2.5% vs. 1.7%; SHR 1.42, 95% CI 1.03–1.95) than the 10,085 matched controls without DVT. They also tended to have a greater risk of all-cause mortality (38.1% vs. 33.1%; hazard ratio [HR] 1.18, 95% CI 1.09–1.27) and systemic thromboembolism (4.2% vs. 2.6%; SHR 1.56, 95% CI 1.22–1.99), respectively. We showed the presence of DVT may be associated with an increased risk of MALEs, major amputation, and thromboembolism, contributing to a higher mortality rate in T2DM.

## Introduction

Diabetes mellitus affects 463 million people worldwide with a global prevalence estimated to be 9.3% in 2019^1^. Diabetes has been associated with a two to fourfold increased risk of critical limb ischemia^2^, and diabetic patients with critical limb ischemia have been reported to have a seven to 15-fold higher risk of undergoing major limb amputation^3,4^ and consequently a high mortality rate^5^. The recent COMPASS^6^ and VOYAGER studies^7^ showed that adding antithrombotic therapy could reduce the incidence of major adverse limb events (MALEs) including amputation. Major amputation due to vascular causes is common in patients with a history of peripheral artery disease (PAD), revascularization and recurrent atrial thrombus^8,9^. Even though arterial and venous thrombosis are generally considered to be separate entities due to differences in vasculature and clot formation. Moreover, body of evidence have shown associations between venous thromboembolism (VTE), including pulmonary embolism (PE) and deep vein thrombosis (DVT), share clusters of CV risks^10^, subsequent arterial cardiovascular events^11–14^ and mortality^15^. Chang et al. were the first to report a significant association between hemostatic varicose and peripheral vascular disease, including DVT and PAD^16^; however, the exact mechanisms have yet to be identified.

Deep vein thrombosis is prevalent in patients with diabetes^17^, and it is regarded to be a prothrombotic state with complications including pulmonary embolism and post-thrombotic syndrome (PTS). Prandoni et al. reported a significantly higher rate of subsequent arterial events in patients with DVT and residual thrombus^18^, and that this could lead to PTS complicated with edema, venous stasis, ulceration, and thrombophlebitis with focal inflammation. Most previous studies have investigated associations between VTE and atherosclerotic cardiovascular disease in the general population, however few studies have focused on potentially life-threatening limb arterial complications in patients with diabetes in this context.

We hypothesized that DVT may be associated with an increased risk of mortality due to a higher rate of subsequent MALEs and major adverse cardiovascular events (MACEs). Therefore, the aim of this study was to assess the influence of DVT on MALEs and MACEs in patients with a new diagnosis of type 2 diabetes.

## Results

### Baseline characteristics

A total of 1,628,675 patients with type 2 diabetes were included, of whom 2020 (0.12%) had a history of DVT hospitalizations before propensity score matching (PSM). The patients in the DVT group were older (66.3 vs. 58.3 years), were more predominantly female (54.3% vs. 47.5%), had higher prevalence rates of several comorbidities (including hypertension, ischemic heart disease, heart failure, atrial fibrillation, PAD, chronic obstructive pulmonary disease, gout, CKD and liver cirrhosis), and a higher Charlson’s Comorbidity Index score than the patients in the non-DVT group. In addition, more were likely to have had a previous stroke and myocardial infarction, and to have more prescriptions for anti-platelet agents, ACEIs/ARBs, beta-blockers, dihydropyridine calcium channel blockers, diuretics, cilostazol and insulin compared to the patients in the non-DVT group. However, the DVT group was less frequently prescribed with metformin or sulfonylurea. After PSM, the distribution of baseline characteristics between groups was well balanced, with all standardized difference absolute values < 0.1 (Table 1).

**Table 1:** Baseline characteristics of the T2DM patients with or without coexisting DVT before and after propensity score matching.

Variables	Data before PSM			Data after PSM		
	DVT (*n* = 2020)	Non-DVT (*n* = 1,629,353)	STD	DVT (*n* = 2017)	Non-DVT (*n* = 10,085)	STD
Age, years	66.3 ± 13.0	58.3 ± 12.7	0.62	66.3 ± 13.0	67.2 ± 12.4	− 0.07
**Age group**						
20–64 years	829 (41.0)	1,110,262 (68.1)	− 0.57	829 (41.1)	3855 (38.2)	0.06
65–74 years	625 (30.9)	354,355 (21.8)	0.21	625 (31.0)	3261 (32.3)	− 0.03
≥ 75 years	566 (28.0)	164,736 (10.1)	0.47	563 (27.9)	2969 (29.4)	− 0.03
**Sex**						
Male	924 (45.7)	856,244 (52.6)	− 0.14	924 (45.8)	4551 (45.1)	0.01
Female	1096 (54.3)	773,109 (47.5)	0.14	1,093 (54.2)	5534 (54.9)	− 0.01
**Comorbidities**						
Hypertension	1240 (61.4)	714,907 (43.9)	0.36	1,239 (61.4)	6455 (64.0)	− 0.05
Dyslipidemia	462 (22.9)	354,324 (21.8)	0.03	462 (22.9)	2361 (23.4)	− 0.01
Ischemic heart disease	488 (24.2)	191,769 (11.8)	0.33	486 (24.1)	2481 (24.6)	− 0.01
Heart failure	261 (12.9)	29,572 (1.8)	0.44	258 (12.8)	1204 (11.9)	0.03
Atrial fibrillation	74 (3.7)	14,095 (0.87)	0.19	74 (3.7)	356 (3.53)	0.01
Peripheral arterial disease	430 (21.3)	61,441 (3.8)	0.55	428 (21.2)	2140 (21.2)	< 0.01
Chronic obstructive pulmonary disease	256 (12.67)	87,880 (5.39)	0.26	256 (12.69)	1258 (12.47)	0.01
Gouty arthritis	247 (12.2)	113,252 (7.0)	0.18	246 (12.2)	1279 (12.7)	− 0.01
**Renal function**						
Normal	1657 (82.0)	1,534,504 (94.2)	− 0.38	1657 (82.2)	8425 (83.5)	− 0.04
Non-dialysis CKD	238 (11.8)	88,325 (5.4)	0.23	238 (11.8)	1229 (12.2)	− 0.01
Dialysis	125 (6.2)	6524 (0.40)	0.33	122 (6.1)	431 (4.27)	0.08
Liver cirrhosis	83 (4.1)	20,165 (1.2)	0.18	82 (4.1)	386 (3.8)	0.01
**History of event**						
Prior stroke	374 (18.5)	90,626 (5.6)	0.41	372 (18.4)	1922 (19.1)	− 0.02
Old myocardial infarction	77 (3.8)	19,978 (1.2)	0.17	77 (3.8)	402 (4.0)	− 0.01
Charlson’s Comorbidity Index score	2.1 ± 1.9	1.1 ± 1.3	0.61	2.1 ± 1.9	2.1 ± 1.9	< 0.01
**Medication within 6 months before the index date**						
Antiplatelet	632 (31.3)	279,257 (17.1)	0.33	631 (31.3)	3280 (32.5)	− 0.03
ACEi/ARB	587 (29.1)	350,719 (21.5)	0.17	586 (29.1)	3094 (30.7)	− 0.04
ß-blockers	475 (23.5)	302,451 (18.6)	0.12	475 (23.6)	2390 (23.7)	< 0.01
DCCB	575 (28.5)	366,165 (22.5)	0.14	574 (28.5)	3024 (30.0)	− 0.03
Diuretics	284 (14.1)	53,728 (3.3)	0.39	283 (14.0)	1372 (13.6)	0.01
Statin	251 (12.4)	177,398 (10.9)	0.05	251 (12.4)	1269 (12.6)	< 0.01
Cilostazol	18 (0.9)	1538 (0.1)	0.11	18 (0.9)	72 (0.7)	0.02
**Lowering hyperglycemia drug at the T2DM diagnostic date**						
Metformin	984 (48.7)	914,705 (56.1)	− 0.15	984 (48.8)	5001 (49.59)	− 0.02
Sulfonylurea	1078 (53.4)	1,058,856 (65.0)	− 0.24	1078 (53.45)	5381 (53.36)	< 0.01
Thiazolidinedione	35 (1.7)	23,483 (1.4)	0.02	35 (1.7)	179 (1.8)	< 0.01
DPP4i	29 (1.4)	11,216 (0.69)	0.07	28 (1.4)	156 (1.6)	− 0.01
Insulin	297 (14.70)	98,799 (6.06)	0.29	295 (14.63)	1,353 (13.42)	0.03
Follow-up, years	5.1 ± 3.7	7.1 ± 4.1	− 0.51	5.1 ± 3.7	5.2 ± 3.9	− 0.02

*T2DM* type II diabetes mellitus, *PSM* propensity score matching, *DVT* deep vein thrombosis, *STD*, standardized difference, *CKD* chronic kidney disease, *ACEi* angiotensin converting enzyme inhibitor; *ARB* angiotensin receptor blocker, *DCCB* dihydropyridine calcium channel blockers, *DPP4i* Dipeptidyl peptidase-4 inhibitor.

### Primary outcomes

**Figure 1:**
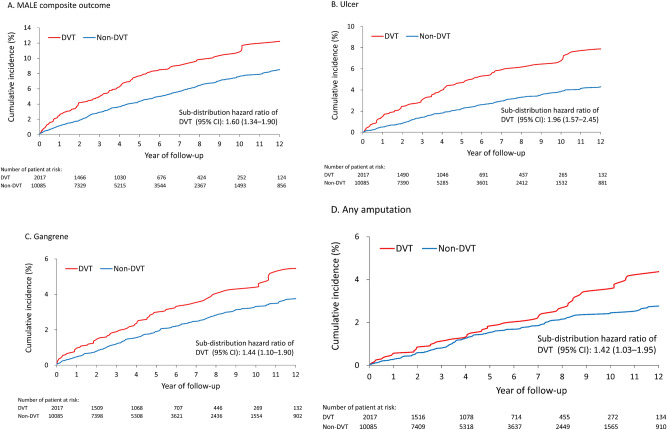
Cumulative incidence function of MALE composite outcome (**A**), foot ulcer (**B**), gangrene (**C**) and any amputation (**D**) of the patients with type 2 diabetes stratified by a history of deep vein thrombosis hospitalization during follow-up in the propensity score-matched cohort.

During a mean follow-up of 5.2 years (standard deviation: 3.9 years), the DVT group had a higher risk of MALE composite outcome (8.4% vs. 5.2%; subdistribution hazard ratio [SHR] 1.60, 95% CI 1.34–1.90) (Fig. 1A). Among the individual components, the DVT group had higher risks of foot ulcer (5.2% vs. 2.6%, SHR 1.96, 95% CI 1.57–2.45), gangrene (3.4% vs. 2.3%, SHR 1.44, 95% CI 1.10–1.90) and amputation (2.5% vs. 1.7%; SHR 1.42, 95% CI 1.03–1.95 Fig. 1B–D; Supplementary Table S2). No difference in the risk of PTA between the DVT and non-DVT groups was observed. Details of incidence density (number of events per 1000 person-years) were shown in Supplementary Table S2.

### Secondary outcomes

**Figure 2:**
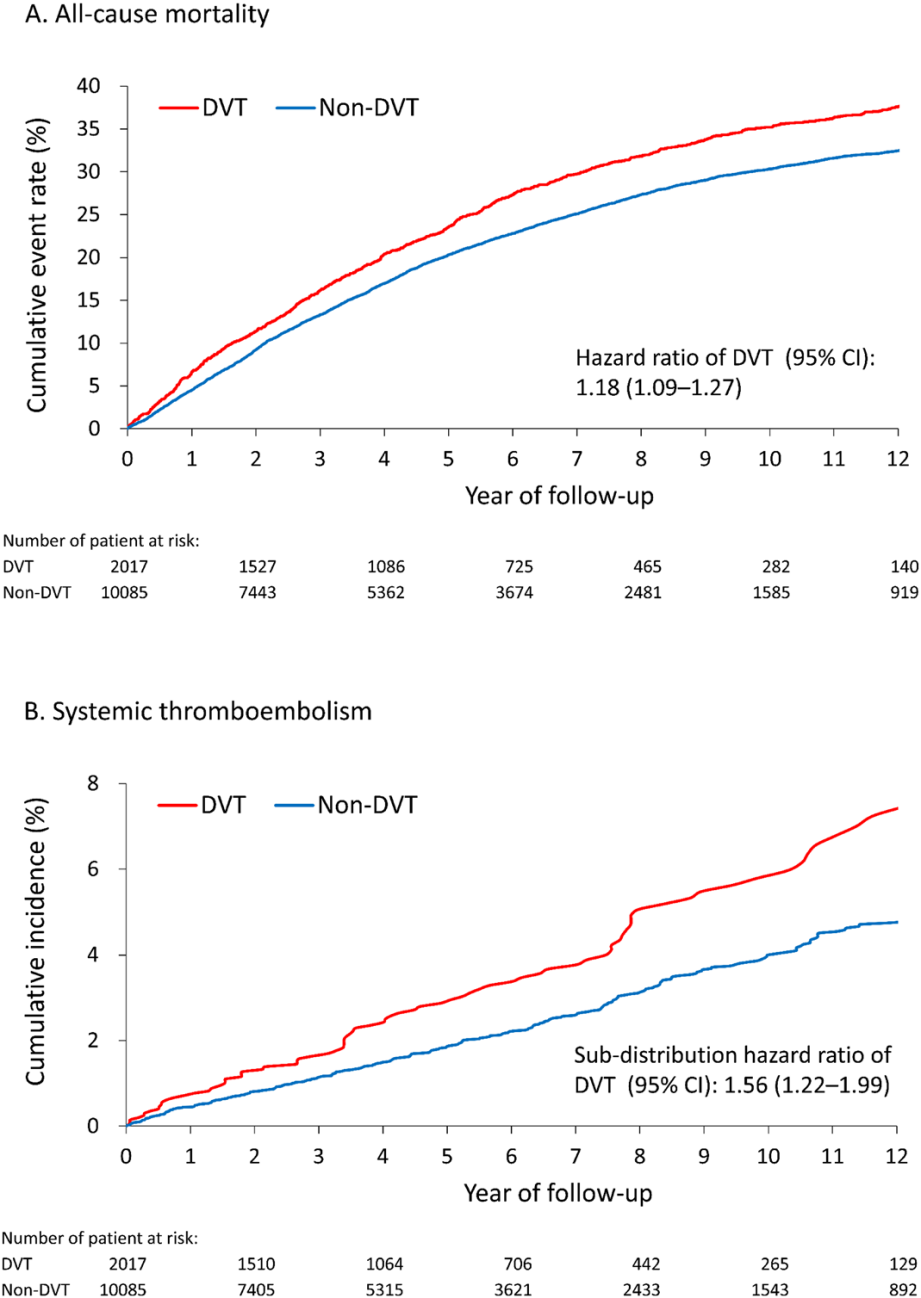
Cumulative event rate of all-cause mortality (**A**) and cumulative incidence function of systemic thromboembolism (**B**) of the patients with type 2 diabetes stratified by a history of deep vein thrombosis hospitalization during follow-up in the propensity score-matched cohort.

The DVT group had a higher risk of all-cause mortality (38.1% vs. 33.1%; hazard ratio [HR] 1.18, 95% CI 1.09–1.27), which was mainly from non-cardiovascular death (22.7% vs. 18.9%; HR 1.22, 95% CI 1.11–1.35) (Fig. 2A). Noticeably, the risk of systemic thromboembolism was also higher in the DVT group than in the non-DVT group (4.2% vs. 2.6%; SHR 1.56, 95% CI 1.22–1.99), especially in lower extremities (3.6% vs. 2.3%; SHR 1.52, 95% CI 1.17–1.98) (Fig. 2B). The risks of cardiovascular death, acute myocardial infarction, ischemic stroke, and heart failure hospitalization were not significantly different between the two groups (Supplementary Table S2).

### Subgroup analysis

We further conducted subgroup analysis of the primary outcomes with statistical significance (including MALEs, amputation, and systemic thromboembolism). The association between a history of DVT and an increased risk of MALEs was more obvious in the patients without CKD (*P* for interaction < 0.05; Supplementary Fig. S3). In contrast, the association between a history of DVT and risks of amputation and systemic thromboembolism were consistent across different subgroups (Supplementary Figs. S2, S3).

## Discussion

This longitudinal cohort study identified significant associations between DVT and MALEs including major amputation and severe wound conditions (ulcer and gangrene) in patients with type 2 diabetes. To the best of our knowledge, this is the first study to show that a history of prior DVT was independently associated with a 1.6-fold higher risk of MALEs and 1.4-fold higher risk of major amputation. These critical events may contribute to functional disability, future mortality, and insurance costs for long-term diabetic care.

In this study, more than 21% of the enrolled diabetic patients with DVT were very elderly (age > 75 years) and had a history of PAD and various comorbidities, both of which tend to be risk factors for MALEs^19^. Female patients have also previously been reported to have worse outcomes after revascularization, however male sex was shown to independently increase the risk of PTS ulcers in the RIETE registry^20^. During long-term follow-up (5.2 years), the average 5-year mortality rate in the current study was 38.1%, which is higher than that in the study of Chang et al. (17.97% at 5 years)^16^. The proportion of patients with diabetes in Chang’s study was 18.43% lower than in our study; however, they found that male patients with diabetes and unprovoked VTE had a higher risk of mortality. Our results suggest that DVT increased the incidence of lower limb complications, arterial thrombosis, and amputation, thereby contributing to poor survival in our patients with type 2 diabetes.

Factors reported to contribute to major limb amputation include wound infection, microvascular dysfunction^3^, and recurrent arterial thrombosis with chronic luminal occlusion^8,9^. Our findings indicate that DVT also has a significant influence on foot ulcer and gangrene in patients with diabetes. The EUCLID trial showed reported an overall rate of major amputation of 8.4% in patients with critical limb ischemia^4^, while the rate of amputation in the DVT group was 2.5% in our study. The rate of revascularization for PAD is low in patients with diabetes and DVT because advanced endovascular interventions are increasingly being used in Taiwan^21^. The COMPASS and VOYAGER studies^6,7^ concluded that thrombosis should not be ignored in PAD, because the addition of anticoagulants can successfully reduce MALEs and mortality. Therefore, systemic thromboembolism may be a major risk factor for MALEs as supported by our results. Even though there were more below the knee amputations than above the knee amputations (38 vs. 17 in the DVT group), the risk of incidental above the knee amputations was still significantly higher (1.97-fold) in the DVT group. Mao et al. reported a high incidence of above the knee amputations in patients with atrial fibrillation receiving PTA, and they concluded that intra-cardiac or up-stream intra-arterial thrombus was the major source^22^. In addition, the patients receiving above the knee amputations had a poor survival rate because of poor limb salvage, functional disability, and ambulance, which is compatible with our analysis. Therefore, DVT may increase the risk of incidental above the knee amputations and mortality in patients with diabetes.

Several studies have reported associations between VTE and cardiovascular disease with cardiovascular risk burden^10^ and arterial thrombosis^11^. However, the influence of DVT on ischemic stroke and myocardial infarction may be inconsistent due to previous studies focusing on unprovoked VTE or pulmonary embolism^16^, in contrast to our study population (diabetic patients with DVT). A 20-year cohort study with a large number of patients reported that DVT and pulmonary embolism were risk factors for acute arterial cardiovascular events; however, the relative risk decreased during 1–5 years^11^. Consuelo et al. further reported that VTE was not associated with subsequent acute myocardial infarction but death^23^. Furthermore, the severity of diabetes, proteinuria, and other CVD-associated risk factors (dyslipidemia and hypertension) have been reported to be major risk factors for future MACEs and cardiac dysfunction. Heart failure is also a known risk factor for DVT^24^, increasing atrial fibrillation burden^26^, physical inactivity and impaired tissue perfusion due to lower cardiac output. Nevertheless, incidental heart failure hospitalizations and cardiovascular events were not prevalent in our DVT group. Taken together, we suggest that the association between DVT and all-cause mortality is closed related to MALEs and systemic thromboembolism rather than traditional cardiovascular risk factors and MACEs. Moreover, our findings suggest that MALEs are mainly due to DVT-associated pathomechanisms, a prothrombotic state and systemic thromboembolism.

Venous thrombosis can induce recurrent thromboembolism, peripheral edema, and inflammation as well as accelerated diabetic atherosclerosis. Those patients with DVT had 2.7–7.1% PTS for 1–3 years observation, and diabetes is also a risk factor of PTS (2.3-fold)^20^. Residual thrombus venous hypertension has been shown to play a central pathogenetic role in PTS, and in turn to cause dilatation of the capillaries and increased endothelial permeability to plasma, proteins, and erythrocytes^25^. The consequences of PTS can result in valve insufficiency, chronic edema, inflammation, hyperpigmentation of the skin, and stasis dermatitis, or even the development of a venous ulcer. Persistent edema has been shown to affect wound healing^27^ and induce an inflammatory cascade as evidenced by increasing levels of CRP (C-Reactive Protein), platelet aggregation^28^, endothelial dysfunction^29^, coagulation disturbance and cytokine activation.

### Strengths and limitations

DVT is less prevalent in Asia than in European countries, and the NHIRD is a good resource for such investigations. However, retrospective surveys have inherent limitations including a lack of data on DVT/PTS severity, echo reports, laboratory findings, body weight, body mass index, lifestyle habits (such as smoking), infected wound condition and physical activity. The RIETE registry observed that the characteristics of obesity and DVT treatment (duration or drugs) failed to predict the risk PTS after acute DVT^20^. Hyperglycemia affects diabetic vascular complications, however, A1c level is not a predictor associated with amputation^5^ or recurrent VTE^30^. Inadequate self-care, living alone without family support, lower social-economic status, and infection with a high white blood cell count have also been shown to be independent risk factors for adverse outcomes in long-term wound care. This may also suggest that we underestimated ulcer/gangrene and severity because less severe foot cases may have been treated at clinics. However, in-hospital records of ulcer and gangrene may be considered to be critical limb events. Thrombophilia and hyperhomocysteinemia have been reported to contribute to both venous and arterial thrombosis^31^. Many factors involving hypercoagulation status are likely to be lost in general practice. We excluded patients with malignancy and autoimmune diseases associated with unprovoked DVT to decrease the effects of hypercoagulation status and recurrent DVT under inadequate therapy.

## Conclusion

We found independent associations between DVT and MALE outcomes including major limb amputation, systemic thromboembolism, and mortality in patients with type 2 diabetes. The impact of a history of DVT highlights the importance of thrombotic prevention in diabetic foot care. We needed a further large-scale randomized control trial to prove the relationship.

## Methods

### Data source

This investigation was a retrospective cohort survey using data from the Taiwan National Health Insurance Research Database (NHIRD)^32^, which is derived from the claims data of the Taiwan National Health Insurance (NHI) program. Taiwan NHI program is a single-payer system covering > 99% of the 23 million residents. All submitted standardized data of healthcare services were prospectively recorded in the NHIRD, including information of demographic characteristics, out-patient medical treatment, operation, procedures, drug prescriptions, medical diagnoses of diseases, details during hospitalization. Diseases are registered according to International Classification of Diseases, Ninth Revision, Clinical Modification (ICD-9-CM) codes, and validation of the NHIRD data is routinely conducted by the NHI Bureau.

### Ethical considerations

The Research Ethics Committee of Chang-Gung Memorial Hospital approved the study protocol (201900915B1). The hospital identification number of each subjects was encrypted and de‑identified to protect their privacy because of retrospective nature of the database study. Therefore, informed consent could be waived for this research according to the announcement of Bureau of NHI, department of Health, National Health Research Institutes. Our research was performed in accordance with relevant guidelines and regulations.

### Patient identification

**Figure 3:**
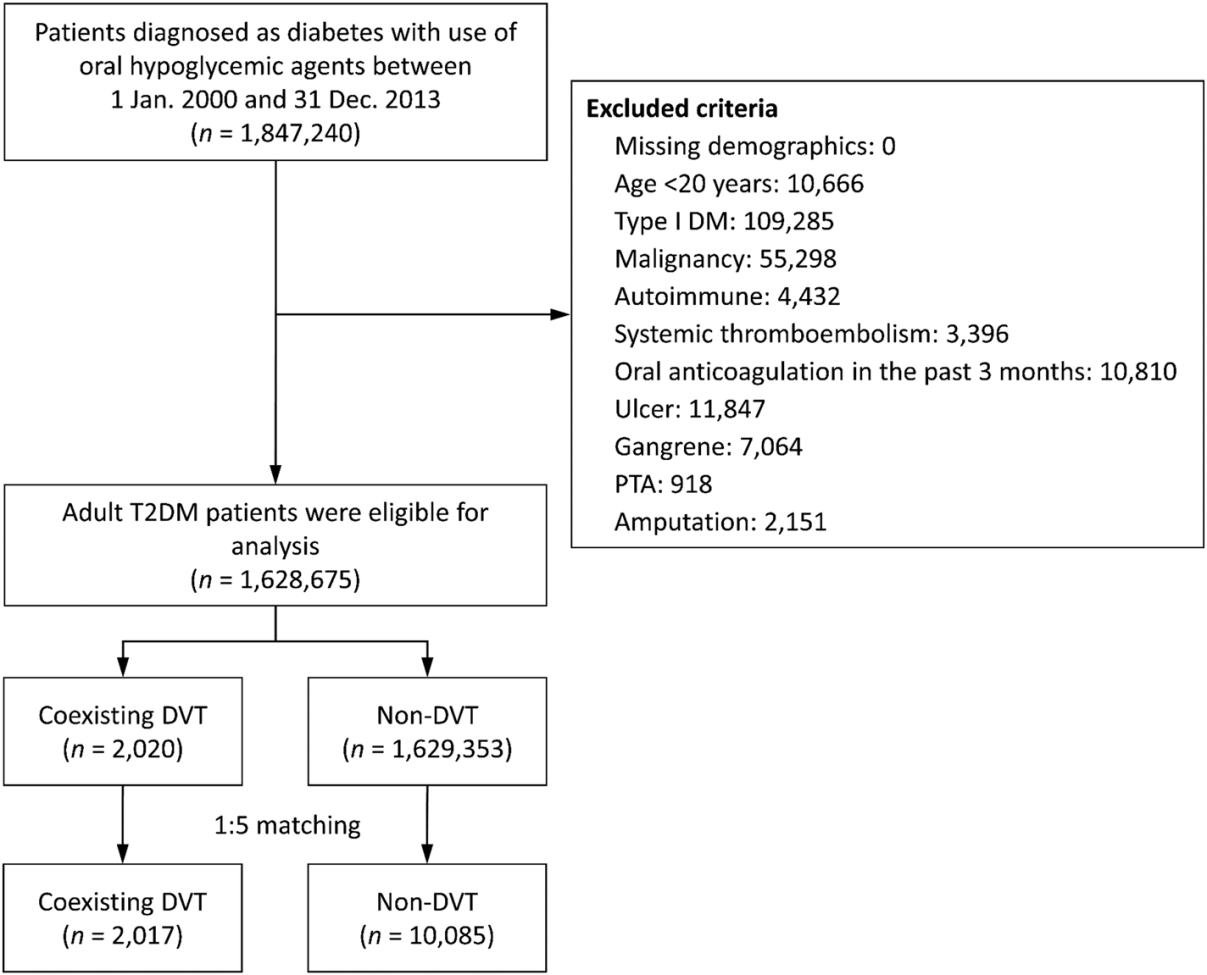
Flowchart of patient inclusion. *DM* diabetes mellitus, *T2DM* type II diabetes mellitus, *DVT* deep vein thrombosis, *PTA* percutaneous transluminal angioplasty.

Patients with a diagnosis of type 2 diabetes in ≥ 5 outpatient visits defined as ICD-9-CM code 250.xx combined with the use of any oral hypoglycemic agents between 2001 and 2013 were identified. The accuracy of a diagnosis of diabetes in the NHIRD has previously been validated^33,34^. The date of the first diagnosis of diabetes was defined as the index date. The patients were divided into two groups: those with coexisting deep vein thrombosis (DVT group), and those without DVT (non-DVT group). A history of DVT was identified using the discharge diagnosis (ICD-9-CM code 453) combined with the use of anticoagulant therapy during an admission before the index date. The accuracy of a diagnosis of DVT has been validated in previous NHIRD studies^35^. The exclusion criteria were: (1) missing demographic data; (2) age < 20 years; (3) type 1 diabetes; (4) history of malignancy, autoimmune diseases, and systemic thromboembolism; (5) history of foot ulcers and gangrene; and (6) medical records of percutaneous transluminal angioplasty (PTA) and amputation. In order to focus on the effect of a history of DVT, patients who had received oral anticoagulant (OAC) treatment 3 months before the index date were also excluded (Fig. 3).

### Covariates

The covariates in this study were age, sex, comorbidities (hypertension, dyslipidemia, ischemic heart disease, heart failure, atrial fibrillation, PAD, chronic obstructive pulmonary disease, gouty arthritis, chronic kidney disease (CKD) or dialysis, liver cirrhosis), Charlson’s Comorbidity Index score, history of events (prior ischemic stroke and previous myocardial infarction), hyperglycemia lowering drugs (metformin, sulfonylurea, thiazolidinedione, dipeptidyl peptidase-4 inhibitors [DPP4is] and insulin) and other medications (antiplatelet, anticoagulation, angiotensin converting enzyme inhibitors/angiotensin receptor blockers [ACEIs/ARBs], ß-blockers, dihydropyridine calcium channel blockers, diuretics, statins and cilostazol). Comorbidities were identified according to at least two clinic visits or any inpatient record in the previous year before the index date and were validated after review by one of the authors^32^. Historical events were identified using any inpatient diagnosis before the index date up to 1997. The use of medications was extracted within 6 months before the index date. All information on medications was extracted from the claims data of outpatient visits or refills for chronic illnesses at the pharmacy using Anatomical Therapeutic Chemical codes or Taiwan NHI reimbursement codes.

### Outcomes

The primary outcome was MALE, which was defined as a composite of foot ulcer, gangrene, PTA, and amputation. Individual MALE components were also investigated. The occurrence of ulcer and gangrene was based on ICD-9-CM diagnostic codes in at least two outpatient visits or any inpatient diagnosis (Supplementary Table S1). The occurrence of PTA and amputation was identified using Taiwan NHI reimbursement codes in inpatient claims data with validation according to a prior study^36^.

The secondary outcomes were all-cause mortality, cardiovascular death, ischemic stroke, acute myocardial infarction, heart failure hospitalization and systemic thromboembolism. All-cause mortality was defined as withdrawal from the NHI program^37^. The definition of cardiovascular death met the criteria of the Standardized Definitions for Cardiovascular and Stroke Endpoint Events in Clinical Trials by the Food and Drug Administration of the United States. The occurrence of ischemic stroke, acute myocardial infarction and heart failure hospitalization was defined as a principal discharge diagnosis of hospitalization. Systemic thromboembolism including arterial thromboembolic occlusion of an extremity or extracranial vital organ such as the kidneys, intestine or spleen. The occurrence of systemic thromboembolism was defined as a principal or secondary diagnosis of hospitalization. Each patient was followed from the index date to the date of an event, date of death, or December 31, 2013, whichever occurred first.

### Statistical analysis

For comparisons of the risk of outcomes between the DVT and non-DVT groups, propensity score matching was performed to reduce possible confounding. The propensity score was the predicted probability to be in one group (i.e., DVT) given the values of covariates using multivariable logistic regression without considering interaction effects. The variables selected to calculate propensity score are listed in Table 1, where the follow-up duration was replaced with the index date. Each subject in the DVT group was matched with five counterparts in the non-DVT group to increase the statistical power. The matching was processed using a greedy nearest neighbor algorithm with a caliper of 0.2 times the standard deviation of the logit of the propensity score, with random matching order and without replacement. The balance after propensity score matching was checked using the absolute value of standardized difference (STD) between the groups, where a value < 0.1 was considered to be a negligible difference.

The risks of fatal outcomes (all-cause mortality and cardiovascular death) between the groups were compared using a Cox proportional hazard model. The incidence rates of non-fatal outcomes (MALEs or acute myocardial infarction) between groups were compared using a Fine and Gray subdistribution hazard model which considered all-cause mortality as a competing risk. The study group (DVT vs. non-DVT) was the only explanatory variable in the survival analyses. Finally, post-hoc subgroup analysis of the primary outcomes with statistical significance (including MALEs, amputation, and systemic thromboembolism) were conducted to assess whether the effect of DVT on outcomes was consistent across different levels of subgroup variables. Selected subgroup variables were age (dichotomized by 75 years), sex, hypertension, dyslipidemia, ischemic heart disease, heart failure, PAD, CKD, prior stroke, and Charlson’s Comorbidity Index score (dichotomized by 2 points).

A two-sided *P* value < 0.05 was considered to be statistically significant, and no adjustment of multiple testing (multiplicity) was made in this study. All statistical analyses were performed using SAS version 9.4 (SAS Institute, Cary, NC), including the ‘psmatch’ procedure for propensity score matching, ‘phreg’ for survival analysis, and a macro of ‘%cif’ to generate the cumulative incidence function under the Fine and Gray subdistribution hazard method.

### Ethics approval and consent to participates

All details in the NHIRD are de-identified to protect privacy, and informed consent could be waived for this retrospective research. The institutional review board of Chang Gung Memorial Hospital had approved the study protocol (201900915B1). Our research had been performed in accordance with relevant guidelines and regulations.

### Consent for publication

All authors have read the manuscript and consent for publication.

## Supplementary Information

Supplementary Legends.

Supplementary Figure S1.

Supplementary Figure S2.

Supplementary Figure S3.

Supplementary Table S1.

Supplementary Table S2.

## Data Availability

The data used to support the findings of this study are available from the corresponding author upon request.
