# Supplementary material for: The effect of deep vein thrombosis on major adverse limb events in diabetic patients: a nationwide retrospective cohort study
Source: Sci Rep. 2021 Apr 13;11:8082. doi: 10.1038/s41598-021-87461-y (PMC8044219; doi:10.1038/s41598-021-87461-y)
Supplement: Supplementary file 1 — Supplementary Legends. [file 41598_2021_87461_MOESM1_ESM.docx]

**Supplementary figures.** Subgroup analysis of MALE (1), amputation (2) and systemic thromboembolism (3).

MALE, major adverse limb event.
