# Supplementary figures and images for: The effect of deep vein thrombosis on major adverse limb events in diabetic patients: a nationwide retrospective cohort study
Source: Sci Rep. 2021 Apr 13;11:8082. doi: 10.1038/s41598-021-87461-y (PMC8044219; doi:10.1038/s41598-021-87461-y)

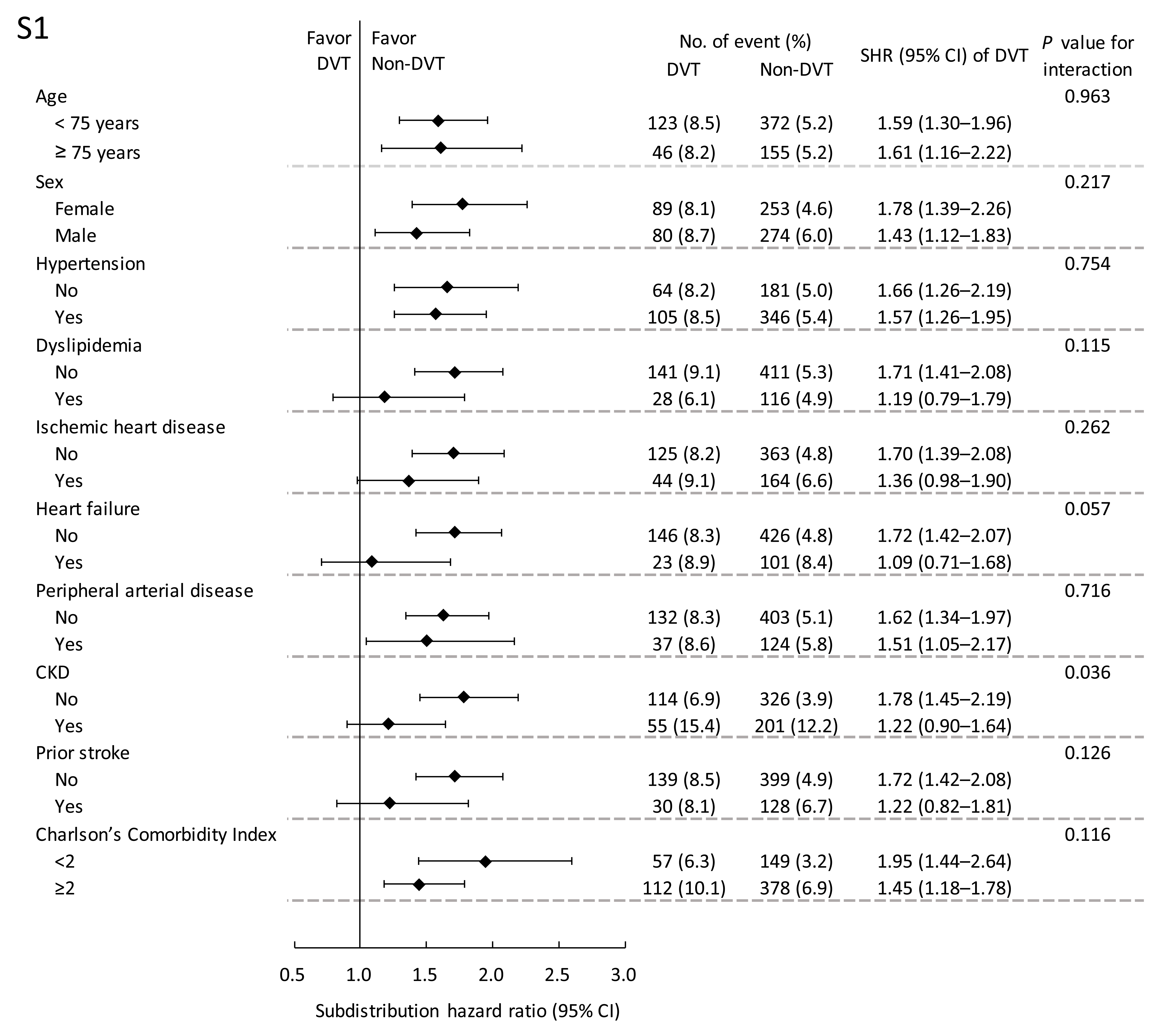

Supplement: Supplementary file 2 — Supplementary Figure S1. [file 41598_2021_87461_MOESM2_ESM.tif]

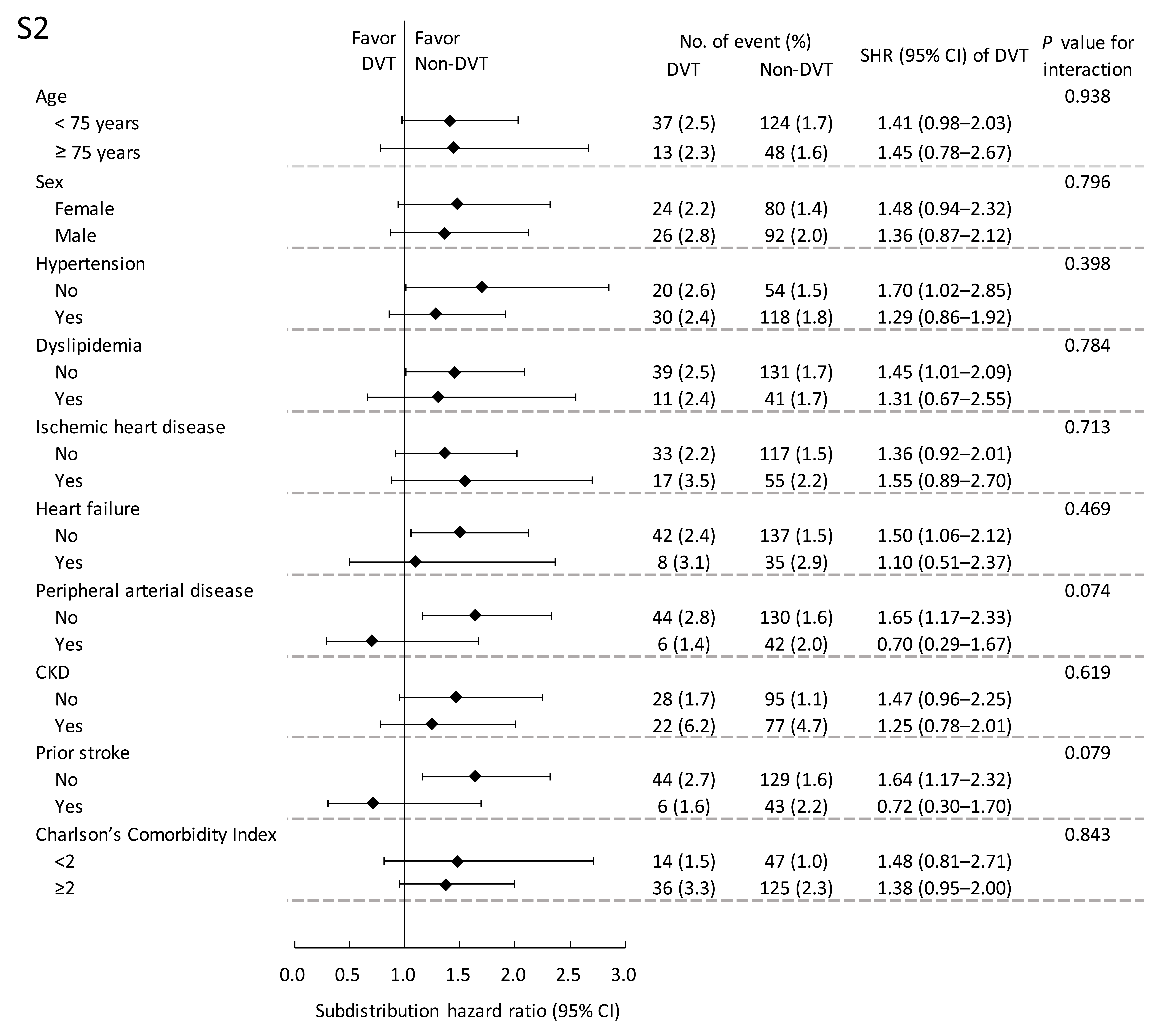

Supplement: Supplementary file 3 — Supplementary Figure S2. [file 41598_2021_87461_MOESM3_ESM.tif]

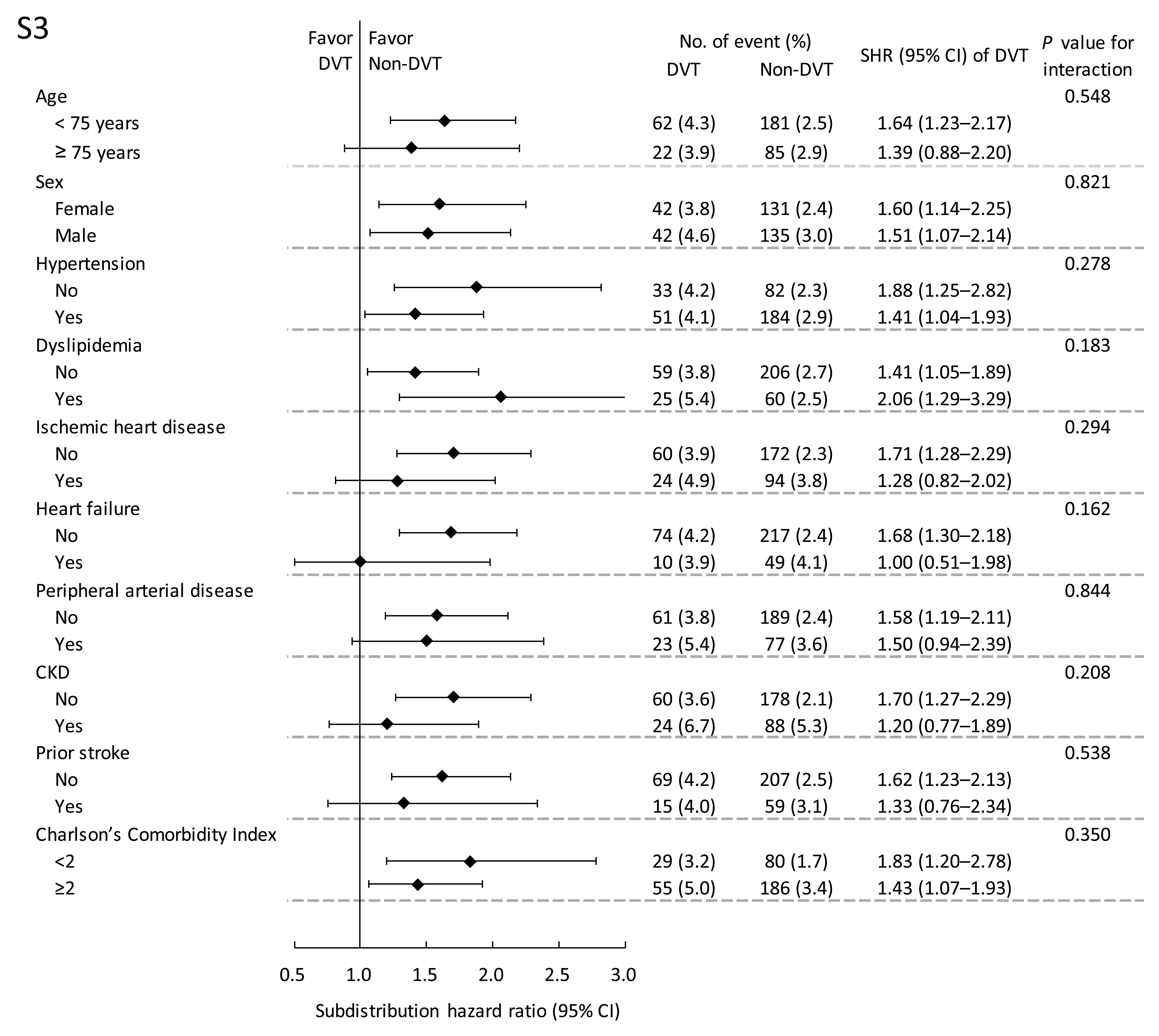

Supplement: Supplementary file 4 — Supplementary Figure S3. [file 41598_2021_87461_MOESM4_ESM.tif]
