# Supplementary material for: The effect of deep vein thrombosis on major adverse limb events in diabetic patients: a nationwide retrospective cohort study
Source: Sci Rep. 2021 Apr 13;11:8082. doi: 10.1038/s41598-021-87461-y (PMC8044219; doi:10.1038/s41598-021-87461-y)
Supplement: Supplementary file 5 — Supplementary Table S1. [file 41598_2021_87461_MOESM5_ESM.docx]

**Supplementary Table S1.** ICD-9-CM code used for diagnosis in the current study

| Diagnosis | ICD-9-CM |
| --- | --- |
| Diabetes mellitus type 2 | 250.xx |
| Diabetes mellitus type 1 | 250.01, 250.03, 250.11, 250.13, 250.21, 250.23, 250.31, 250.33, 250.41, 250.43, 250.51, 250.53, 250.61, 250.63, 250..71, 250.73, 250.81, 250.83, 250.91, 250.93 |
| Malignancy | 140.xx–208.xx (Catastrophic illness card) |
| Autoimmune disease | 710.xx, 714.xx, 555.xx, 556.xx (Catastrophic illness card) |
| Systemic thromboembolism | 444.22, 444.81, 444.21, 362.30, 362.34, 444.89, 557.0, 444.9x |
| Ulcer | 440.23, 707.1x |
| Gangrene | 785.4, 440.24 |
| Deep vein thrombosis | 453.xx |
| Hypertension | 401.xx–405.xx |
| Dyslipidemia | 272.xx |
| Ischemic heart disease | 410.xx-414.xx |
| Heart failure | 428.xx |
| Atrial fibrillation | 427.31 |
| Peripheral arterial disease | 440.0x, 440.2x, 440.3x, 440.8x, 440.9x, 443.xx, 444.0x, 444.22, 444.8x, 447.8x, and 447.9x |
| Chronic obstructive pulmonary disease | 491.xx, 492.xx, 496.xx |
| Gouty arthritis | 274.xx |
| Liver cirrhosis | 571.5, 571.6, 571.2 |
| Prior stroke | 430.xx–437.xx |
| Old myocardial infarction | 410.xx, 412.xx |

ICD-9-CM, International Classification of Diseases, Ninth Revision, Clinical Modification.
