# Supplementary material for: The effect of deep vein thrombosis on major adverse limb events in diabetic patients: a nationwide retrospective cohort study
Source: Sci Rep. 2021 Apr 13;11:8082. doi: 10.1038/s41598-021-87461-y (PMC8044219; doi:10.1038/s41598-021-87461-y)
Supplement: Supplementary file 6 — Supplementary Table S2. [file 41598_2021_87461_MOESM6_ESM.docx]

**Supplementary Table S2.** Follow-up outcomes of the T2DM patients with or without coexisting DVT in the propensity score matched cohort

|  | DVT (*n* = 2,017) | |  | Non-DVT (*n* = 10,085) | |  |  |
| --- | --- | --- | --- | --- | --- | --- | --- |
| Outcome | No. of  event (%) | ID (95% CI)* |  | No. of  event (%) | ID (95% CI)* | HR or SHR for DVT  (95% CI) | *P* value |
| Primary outcome: MALE related |  |  |  |  |  |  |  |
| Ulcer | 105 (5.2) | 10.5 (8.5–12.5) |  | 265 (2.6) | 5.1 (4.5–5.8) | 1.96 (1.57–2.45) | <0.001 |
| Gangrene | 68 (3.4) | 6.7 (5.1–8.3) |  | 231 (2.3) | 4.5 (3.9–5.0) | 1.44 (1.10–1.90) | 0.009 |
| Percutaneous transluminal angioplasty | 33 (1.6) | 3.2 (2.1–4.3) |  | 133 (1.3) | 2.6 (2.1–3.0) | 1.21 (0.83–1.76) | 0.315 |
| Amputation |  |  |  |  |  |  |  |
| Above knee | 17 (0.84) | 1.7 (0.87–2.4) |  | 42 (0.42) | 0.80 (0.56–1.1) | 1.97 (1.12–3.47) | 0.019 |
| Below knee | 38 (1.9) | 3.7 (2.5–4.9) |  | 153 (1.5) | 2.9 (2.5–3.4) | 1.21 (0.85–1.73) | 0.301 |
| Any amputation | 50 (2.5) | 4.9 (3.5–6.3) |  | 172 (1.7) | 3.3 (2.8–3.8) | 1.42 (1.03–1.95) | 0.031 |
| MALE composite outcome | 169 (8.4) | 17.3 (14.7–19.9) |  | 527 (5.2) | 10.3 (9.5–11.2) | 1.60 (1.34–1.90) | <0.001 |
| Secondary outcome |  |  |  |  |  |  |  |
| All-cause mortality | 768 (38.1) | 74.4 (69.1–79.7) |  | 3,338 (33.1) | 63.7 (61.5–65.9) | 1.18 (1.09–1.27) | <0.001 |
| Cardiovascular death | 311 (15.4) | 30.1 (26.8–33.5) |  | 1,430 (14.2) | 27.3 (25.9–28.7) | 1.12 (0.99–1.26) | 0.064 |
| Non-cardiovascular death | 457 (22.7) | 44.3 (40.2–48.3) |  | 1,908 (18.9) | 36.4 (34.8–38.1) | 1.22 (1.11–1.35) | <0.001 |
| Ischemic stroke | 219 (10.9) | 22.9 (19.9–25.9) |  | 1,081 (10.7) | 22.2 (20.9–23.5) | 0.98 (0.85–1.13) | 0.768 |
| Acute myocardial infarction | 50 (2.5) | 4.9 (3.5–6.2) |  | 267 (2.7) | 5.2 (4.5–5.8) | 0.91 (0.67–1.23) | 0.528 |
| Systemic thromboembolism | 84 (4.2) | 8.3 (6.5–10.1) |  | 266 (2.6) | 5.1 (4.5–5.8) | 1.56 (1.22–1.99) | <0.001 |
| Lower extremity | 73 (3.6) | 7.2 (5.6–8.9) |  | 236 (2.3) | 4.5 (4.0–5.1) | 1.52 (1.17–1.98) | 0.002 |
| Non-lower extremity | 15 (0.74) | 1.5 (0.72–2.2) |  | 42 (0.42) | 0.80 (0.56–1.1) | 1.74 (0.97–3.12) | 0.063 |
| Heart failure hospitalization | 136 (6.7) | 13.6 (11.3–15.9) |  | 640 (6.4) | 12.6 (11.6–13.6) | 1.03 (0.86–1.24) | 0.727 |

Abbreviations: T2DM, type II diabetes mellitus; DVT, deep vein thrombosis; ID, incidence density; HR, hazard ratio; SHR, subdistribution hazard ratio; CI, confidence interval; MALE, major adverse limb events;

Data were presented as frequency (percentage);

* Number of event per 1,000 person-years.
